# Simulation of a Temperature-Compensated Voltage Sensor Based on Photonic Crystal Fiber Infiltrated with Liquid Crystal and Ethanol

**DOI:** 10.3390/s22176374

**Published:** 2022-08-24

**Authors:** Wei-Lin Wang, Qiang Liu, Zhao-Yang Liu, Qiang Wu, Yong-Qing Fu

**Affiliations:** 1 School of Control Engineering, Northeastern University at Qinhuangdao, Qinhuangdao 066004, China; 2 Hebei Key Laboratory of Micro-Nano Precision Optical Sensing and Measurement Technology, Qinhuangdao 066004, China; 3 College of Precision Instruments and Optoelectronic Engineering, Tianjin University, Tianjin 300072, China; 4 Faculty of Engineering and Environment, Northumbria University, Newcastle upon Tyne NE1 8ST, UK

**Keywords:** photonic crystal fiber, voltage sensor, liquid crystal, mode coupling, finite element method, temperature compensation

## Abstract

A simulated design for a temperature-compensated voltage sensor based on photonic crystal fiber (PCF) infiltrated with liquid crystal and ethanol is presented in this paper. The holes distributed across the transverse section of the PCF provide two channels for mode coupling between the liquid crystal or ethanol and the fiber core. The couplings are both calculated accurately and explored theoretically using the finite element method (FEM). The influence of voltage on the alignment of the liquid crystal molecules and confinement loss of the fiber mode are studied. Liquid crystal molecules rotate which changes its properties as the voltage changes. As the characteristics of the liquid crystal will be affected by temperature, therefore, we further fill using ethanol, which is merely sensitive to temperature, into one hole of the PCF to realize temperature compensation. The simulated results show that the sensitivity is up to 1.29977 nm/V with the temperature of 25 °C when the voltage ranges from 365 to 565 V. The standard deviation of the wavelength difference is less than 2 nm within the temperature adjustment from 25 to 50 °C for temperature compensation. The impacts of the construction parameters of the PCF on sensing performances of this voltage sensor are also analyzed in this paper.

## 1. Introduction

In this high-speed information age, more sensors with fast transmission, large capacity, high electromagnetic interference resistance, and compact size are needed. Due to these strengths, the optical fiber sensing technology has rapidly developed, bringing more opportunities for optical communication, optical sensing, and many other fields. In the last 20 years, a novel type of optic fiber named photonic crystal fiber (PCF) has been emerging rapidly which is generally made up of just one medium. The microstructure cladding of PCFs is lined with a periodic series of wavelength-scaled air holes that are densely packed in the two-dimensional direction while maintaining the same axial structure [1]. Compared with other conventional optical fibers, the diversity in the structure design of the PCFs is particularly abundant [2]. As the substrate materials are not environmentally sensitive, the different environmentally sensitive materials are filled into these air holes to create sensors with high sensitivity.

In 1954, J.R. Pierce first proposed the concept of mode coupling in the electromagnetic field during the discussion of the application of traveling wave tubes [3]. Later in the 1970s, A.W. Snyder [4], D. Marcuse [5], and A.Yariv [6] introduced the mode-coupling theory into the optical waveguide. Ever since, the mode-coupling theory has been widely used in the optical waveguide to make optical devices, such as waveguide directional couplers [7,8,9], fiber optic couplers [10], distributed feedback lasers [11], and so on. The mode coupling effect in optical fibers produces a peculiar optical phenomenon and presents excellent optical properties. In recent years, special optical fibers based on the mode coupling effect show unique advantages in confinement loss, dispersion, and nonlinearity, and have been extensively adopted for a large collection of application areas in optical fiber communication and optoelectronic systems. Particularly, mode coupling theory gradually gains attention in fiber sensors. Filling sensitive materials into PCF brings diverse structures, in which another mode is exhibited and the coupling resonance with the fiber mode occurs when waveguide phase matches.

Liquid crystal (LC), as a material sensitive to temperature, electric field, and magnetic field, has been gradually applied in photonic crystal fiber sensors. In addition, LC has birefringent optical properties like crystal [12], which makes great contributions to the sensor sensitivity. Larsen and Bjarklev et al. from the Danish Institute of Technology were the first to fill liquid crystal into photonic crystal fibers to make fiber sensors. Through controlling the temperature and electric field, they proposed the concept of optical switch [13]. In 2009, Lei Wei developed an LC-filled PBG-PCF using negative permittivity, which could realize electronic and temperature control tuning. At the voltage value of 200 Vrms, the control temperature range was 20–80 °C and the bandgap change was 396 nm [14]. In 2015, Hu and Fu et al. put forward an all-fiber Mach–Zehnder interferometer on the basis of liquid crystal filling. They filled the core of a hollow PCF with LC and welded single-mode optical fibers on both ends of the filled PBG-PCF to make a Mach–Zehnder interferometer, reaching a temperature sensitivity of up to −1.55 nm/°C [15]. It can be seen that the LC-filled PCFs could expand the application function of such fibers. In this paper, LC is infiltrated into one hole of the PCF to realize the voltage sensor by the coupling between the LC mode and fiber mode.

Voltage sensors are widely applied in power distribution systems, cables, electric machinery, and so on. In order to develop voltage sensors with high performances, research on the electro-optic effect [16,17,18], electro-strictive effect [19,20,21], and so on, are generally studied. Fiber voltage sensors have been widely put into application in various sensing sectors due to numerous distinctive qualities, including small weight, rapid reaction, and capability of remote operation. As the voltage surpasses the threshold, the LC molecules inside the air holes spin towards the presence of an electric field. Plenty of studies have been devoted to development of electric optical fiber devices filled with nematic liquid crystal (NLC). Haakestad et al. filled liquid crystal into a refractive-index-directed PCF to obtain a bandgap-guided fiber with a millisecond electrical reaction time [22]. Ertman et al. injected LC into a high-index PCF, enabling them to alter the phase birefringence adjustment at 0~2 × 10^−4^ [23]. Du et al. developed an electrically tunable filter in which the LC was loaded into PCF in a Sagnac loop with a tuning range of roughly 26 nm, reaching a sensitivity of 0.53 nm/V [24]. Huang et al. developed an electro-optical modulation established on the resonance principle, using LC injected into a PCF. The modulation range is between 1414 and around 1700 nm, reaching a sensitivity of 5.594 nm/V [25]. Our research group has undertaken some research on the electrical sensing characteristics based on PCF infiltrated with LC [26,27,28]. However, the temperature stability and reliability are not studied adequately, and the measurement range is not wide enough. Therefore, the voltage sensors based on PCF filled with LC are worth pursuing and in urgent need of development.

In this paper, the finite element method (FEM) based on the COMSOL Multiphysics software is applied to analyze the sensing performances of the PCF infiltrated with functional materials. The fiber mode and LC mode couple with each other as the phase matches. As we change the voltage of ambient environment, ranging from 365 to 565 V, the confinement loss and the resonance wavelength change simultaneously by which voltage can be detected. However, the characteristics of LC would be affected by temperature which would interference with sensor performance. Therefore, ethanol, which is merely sensitive to temperature, is infiltrated into one hole of the PCF to realize temperature compensation. Meanwhile, the geometrical parameters of the PCF on the sensing characteristics are studied.

## 2. Structure of PCF and Material Parameters

**Figure 1:**
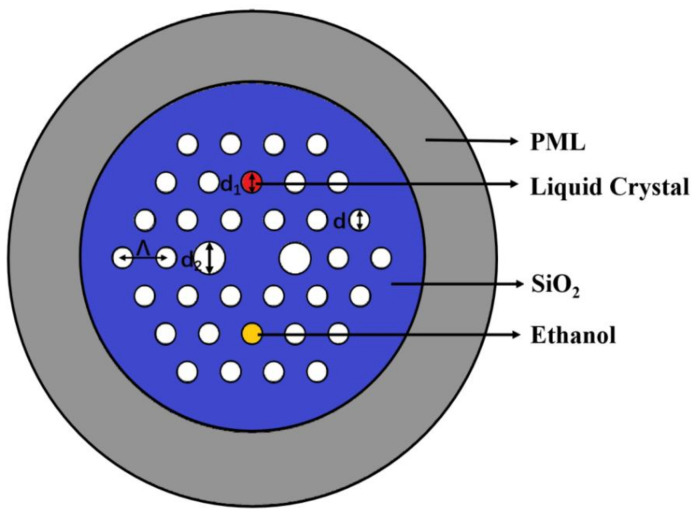
The transverse section of the proposed PCF infiltrated with liquid crystal and ethanol.

The transverse section of proposed PCF infiltrated with LC and ethanol in this paper is clearly depicted in Figure 1. The red hole with nematic LC filled in has a diameter of d_1_ = 1.4 μm. The two larger stomata in the x-direction have a diameter of d_2_ = 2.2 μm and other stomata are denoted by d = 1.4 μm and have a refractive index of 1.0 by which the PCF is a polarization maintaining fiber and possesses high birefringence. The yellow hole in the symmetric position is filled with ethanol, which is utilized to compensate for temperature drift problems. The triangular-lattice distribution is chosen whose spacing is characterized by Λ = 3 μm. Radiation absorbers based on border conditions of the perfectly matched layer (PML) are utilized for completely assimilating the radiant vitality within the external area.

This structure of the PCF infiltrated with functional materials is designed to study its sensing characteristics. The preparation technology of the polarization maintaining PCF is very mature with the stacking method; therefore, it is not difficult to obtain the structure of this PCF. According to the existing filling methods, the capillary method is the most commonly used to fill LC into the air holes. In this paper, we selectively fill LC, which can be a little more difficult than full filling. We can use the laser perforation method [29]. A single-mode fiber is first fused to the PCF, then we use a laser to cut the single-mode fiber. Next, a femtosecond laser is used to punch holes in the cross-section of the single-mode fiber, so that the holes made are in series with the air holes to be filled. Thus, we can achieve selective filling based on the liquid full-filled method.

The backing material of the PCF is pure quartz (SiO_2_), the dispersion of which is defined by the Sellimeier Equation as shown in Equation (1) [30]:

(1) $$n^{2}=1+\frac{0.6961663\lambda^{2}}{\lambda^{2}-{\left(0.0684043\right)}^{2}}+\frac{0.4079426\lambda^{2}}{\lambda^{2}-{\left(0.1162414\right)}^{2}}+\frac{0.8974794\lambda^{2}}{\lambda^{2}-{\left(9.896161\right)}^{2}}$$

In this structure, the nematic LC E_7_, which is filled in the red hole, is an anisotropic liquid. LC E_7_ forms a rod-like structure, which can be described in terms of ordinary refractive index $n_{o}$ and extraordinary refractive index $n_{e}$. $n_{o}$ and $n_{e}$ are calculated using Equation (2) [31]:

(2) $$n_{o,e}=A_{o,e}+\frac{B_{o,e}}{\lambda^{2}}+\frac{C_{o,e}}{\lambda^{4}}$$

where *A_o_* = 1.4994, *B_o_* = 0.007, *C_o_* = 0.0004, *A_e_* = 1.6933, *B_e_* = 0.0078, and *C_e_* = 0.0028 when the temperature is 25 °C. Thus, the specific arrangement of the refractive index is n_LC_ = diag[*n_o_,n_o_,n_e_*].The basic parameters and values of E_7_ are shown in Table 1. The refractive index of LC is higher than that of the background material silica, while the light is introduced into the core of the special fiber, the fiber core is surrounded by air holes with low refractive index, therefore, it can still guide light using total internal reflection. The mode resonance will occur as the phase matching condition is satisfied and the sensing mechanism is based on the coupling between the fiber-core mode and LC mode. The simulation results show that the light-guiding mechanism is not modified by the infiltrated materials.

**Figure 2:**
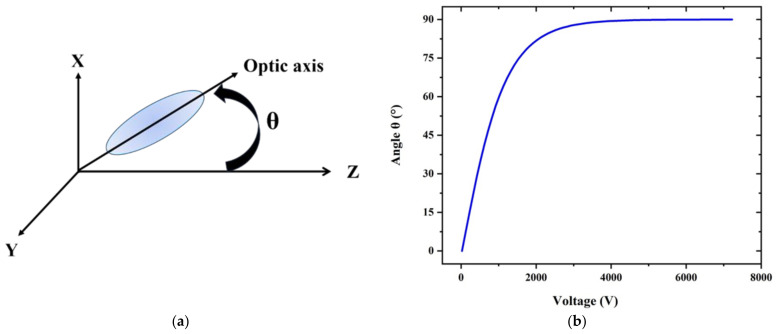
(**a**) The arrangement of liquid crystal molecules under an external electric field. (**b**) The relationship between the rotation angle of LC and ambient voltage.

Without any electric field, the LC molecules filling the PCF holes are arranged in a planar pattern with their orientation parallel to the fiber axis. As the electric field is exerted, LC molecules will rotate. The angle of rotation can be calculated using Equation (3) [32]:

(3) $$\theta =\left\{\;\begin{array}{c} 0\;\;\;\;\;\;\;\;\;\;\;\;\;\;\;\;\;\;\;\;\;\;\;\;\;\;\;\;\;\;\;\;\;\;\;\;\;\;\;\;\;\;\;\;\;\;\;\;\;\;\;\;\;\;\;\;\;\;\;\;\;\;\;\;\;\;\;\;\;\;\;\;\;\;\;\;\;V\leq V_{C}\\ \frac{\pi }{2}-2\tan^{-1}\left[exp\left(-\frac{V-V_{C}}{30V_{C}}\right)\right]\;\;\;\;\;\;\;\;\;\;\;\;\;\;\;\;\;\;\;\;\;\;\;\;\;\;\;V>V_{C} \end{array}\right.$$

where $V_{C}$ denotes the threshold voltage. Once the threshold voltage is reached, the molecules start to revolve, as Figure 2a depicts. When the voltage is at its saturation value, the rotation angle θ approaches 90°. Figure 2b illustrates the relation between the rotation angle and the ambient voltage.

Threshold electric fields could be characterized by [22]:

(4) $$E_{C}=\frac{\pi }{d}\sqrt{\frac{K11}{\Delta \varepsilon }}$$

where $\Delta \varepsilon =\varepsilon_{∥}-\varepsilon_{⊥}$, d is the diameter of the filled hole, K11 indicates a splay elastic constant of 11.1 pN, the dielectric constant of $\varepsilon_{⊥}$ = 5.2ε_0_ and $\;\varepsilon_{∥}$ = 19.3ε_0_ on the ordinary *y*-axis and extraordinary *y*-axis, respectively, and an electric field with a frequency of 1 kHz is attached. The threshold voltage of LC E_7_ is calculated to be 33.83 V. The voltage range from 365 to 565 V is considered in this paper. The permittivity of the diagonal tensor is described by ε = diag[$T_{xx},T_{yy},\;T_{zz}$] [23], where

(5) $$T_{xx}=n_{o}^{2}$$

(6) $$T_{yy}=\cos^{2}\theta n_{o}^{2}+sin^{2}\theta n_{e}^{2}$$

(7) $$T_{zz}=\cos^{2}\theta n_{e}^{2}+sin^{2}\theta n_{o}^{2}$$

As the voltage becomes higher, the rotation angle θ also becomes larger so we can find that *T_xx_* is steady, *T_yy_* increases while *T_zz_* decreases. It means that the refractive index of the mode in the y direction increases.

## 3. Results and Discussion

The LC mode and the fiber mode can produce resonance at one wavelength point as the phase fitting condition is satisfied and part of the energy of the fiber mode is captured by the LC mode, therefore, the energy of the fiber mode decreases, reflecting the maximum confinement loss value. As the voltage in the external environment changes, the resonant wavelength would be different. By detecting the offset of the wavelength at the point of maximum losses, the voltage changes can be deduced backwards. The confinement loss of the fiber mode is defined by the equation below [33]:

(8) $$\alpha =8.686\times \frac{2\pi }{\lambda }Imn_{eff}\times 10^{6}$$

where λ refers to the operating wavelength in μm, $Im\left(n_{eff}\right)$ represents the imaginary portion of effective refractive index, and the losses are calculated using the unit of dB/m.

**Figure 3:**
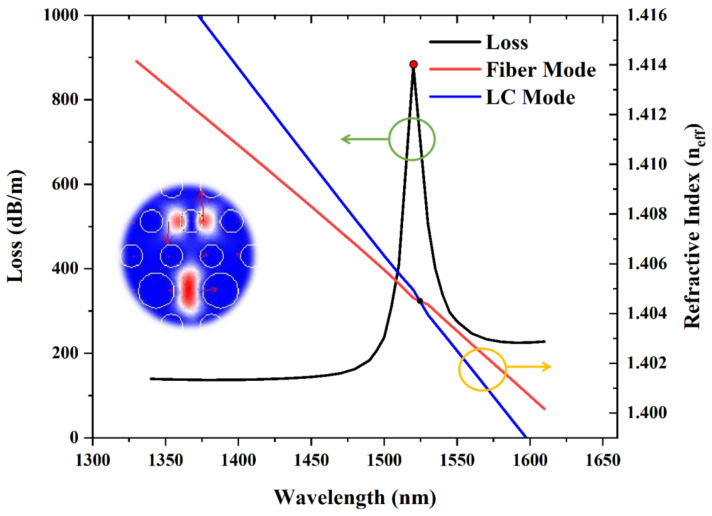
The black curve with green circle for the confinement loss of the fiber mode in the x-polarized direction and the red and blue lines with yellow circle for the variations in the real portion of effective refractive index of the fiber mode and LC defect mode as the external voltage is 465 V. The resonance wavelength is 1523 nm.

Figure 3 shows the confinement loss of the fiber mode and the real portion of the effective refractive index of the fiber mode and LC defect mode. We find the refractive indices of both the fiber mode and LC defect mode diminishes as the wavelength grows, while that of the LC defect mode decreases faster, and the two lines intersect at a point. The highest confinement loss is obtained at the resonance wavelength of 1523 nm with an external voltage of 465 V. The fiber mode and LC defect mode couple with each other the strongest when the blue line and the red line are crossed. Here, the peak of the confinement loss of the fiber mode in red spot appears at 880.297 dB/m. This is a spot when the corresponding resonant wavelength is 1523 nm. From then on, they transform into the opponent mode in the x-polarized orientation. The energy of the fiber mode becomes a part of the LC mode instead. The coupling resonance affects the mode field distribution. This is called complete coupling.

**Figure 4:**
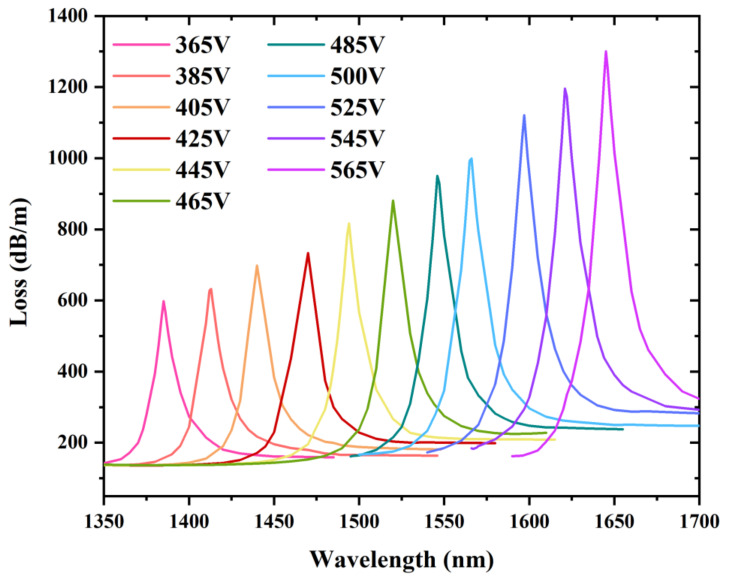
The shift of the loss spectrum of the fiber mode in the x-polarized direction as the voltage increases from 365 to 565 V.

Figure 4 shows the confinement loss spectrum of the fiber mode at varying external voltages, ranging from 365 to 565 V, where the temperature is 25 °C. The resonant wavelength for both the fiber mode and LC defect mode shifts along the long wavelength direction, resulting in a red shift. Moreover, it is clearly seen that the intensities of the confinement loss at resonance wavelengths rise with the external electric field increasing. Thus, this mechanism can be effectively utilized for sensing. As the length of the PCF filled with LC is 1 cm, the transmittance is about −10 dB at the resonance wavelength when the confinement loss is 1000 dB/m. If the length of the PCF filled with LC continues to increase, the transmittance will be lower. The length of the PCF filled with LC should be selected according to the practical applications and photoelectric detector.

**Figure 5:**
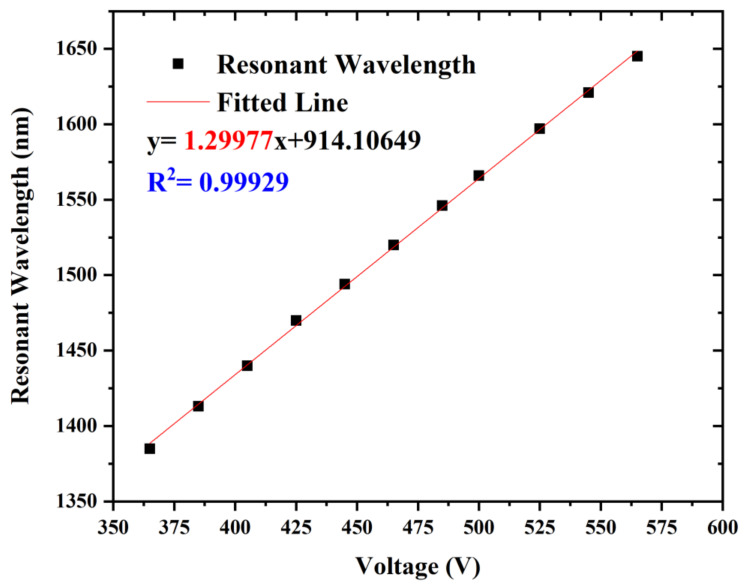
The resonant wavelength of the loss spectrum depending on the external voltage and its fitted line.

The resonant wavelength of the loss spectrum depending on the external voltage is depicted in the Figure 5, the equation of the fitted line is y = 1.29977x + 914.10649, and R^2^ = 0.99929, which means that this PCF voltage sensor possesses a sensitivity of 1.29977 nm/V. Based on the data above, the proposed voltage sensor is highly competent in the voltage measurement field and has a high degree of linearity. Hence, we can easily detect the change in external voltage according to the offset of the resonant wavelength. We chose a higher voltage range from 365 to 565 V. At this range, plenty of industrial settings can be applied such as generation for hydropower stations, power module packages in the automotive industry, and network power supply in railway engineering.

Given the future experiment of this sensing method, the corresponding experimental apparatus were introduced in our previous papers [27,28]. A broadband source and optical spectrum analyzer will be utilized. The ITO glass connected to AC source is utilized to provide a positive polarity voltage waveform with a 1 kHz sinusoidal electrical signal. A polarization controller is utilized to modulate the polarization state of the light. Fiber sensors belong to passive devices. As the functional materials are filled into PCF, strong interaction and mutual coupling occur between light and matter by which the detection precision can be improved.

## 4. Temperature Compensation

Considering the Poisson effect of the fiber, optical fiber sensors often have severe temperature cross-sensitivity. Furthermore, the refractive index (RI) of LC is also sensitive to temperature changing; the corresponding coefficients A, B, and C in Equation (2) at different temperatures are obtained in [31]. Ethanol is a heat-sensitive material and it is particularly sensitive to temperature, whose sensitivity coefficient can reach −4 × 10^−4^ RIU/°C. The RI of ethanol can be characterized by Equation (9):

(9) $$n=1.36242-4\times 10^{-4}\times \left(T-291.5\right)$$

where *T* represents the temperature in Kelvin.

Since the RI of ethanol itself is quite sensitive to temperature changes of the external environment, filling ethanol into the holes of PCF can achieve great sensitivity to temperature measurements. In the structure of the proposed sensor, the LC is influenced by both temperature and voltage, while the ethanol is only influenced by temperature. When LC and ethanol are specifically penetrated into two air holes of PCF, we can detect the corresponding resonant wavelength. The resonant wavelength of the LC-filled fiber shifts beneath diverse electric fields, but the electric fields have no effect on the ethanol-filled one [34]. Nevertheless, the impacts of temperature on the two resonance wavelengths are almost equivalent, leading to the same wavelength shift. Thus, the temperature cross-sensitivity can be removed by computing the discrepancy between the two resonant wavelengths. According to the analysis above, we tried to fill ethanol in the yellow hole in Figure 1, which is in the symmetric position of the LC. Thus, we further present a temperature-compensated voltage sensor based on the differential mode resonance.

**Figure 6:**
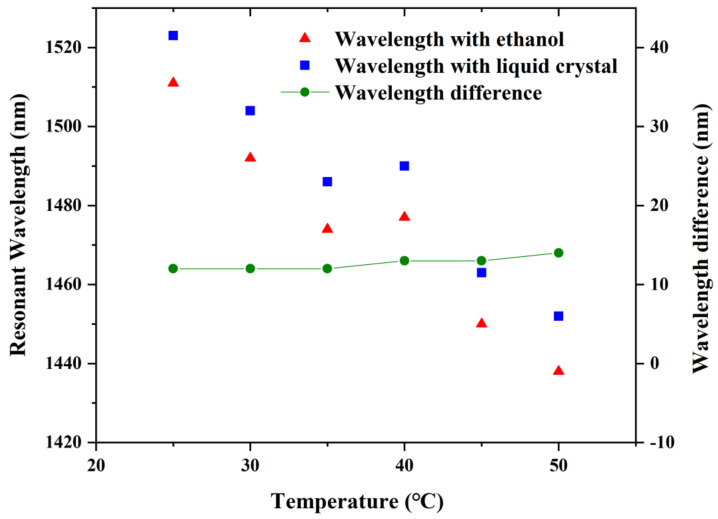
Wavelength shifts of the ethanol- and LC-infiltrated fiber, and wavelength difference between the two resonant wavelengths at the maximum losses.

The resonant wavelengths shift to the shorter wavelength when the temperature grows, as shown in Figure 6. This is because the refractive indexes of LC mode or ethanol mode decrease with increasing temperature, while the refractive index of the fiber-core mode remains unchanged which leads to the phase matching points shifting to shorter wavelengths. Meanwhile, it can be obviously noticed that the wavelength difference is almost settled, where the surrounding voltage is 465 V. The standard deviation in wavelength difference is less than 2 nm within the temperature adjustment from 25 to 50 °C. The temperature sensitivity is calculated as −3.8 nm/°C. As a result, the proposed sensor can make a great compensation for the temperature variations, thus, conquering the temperature cross-sensitivity of the LC-filled fiber optic voltage sensor.

## 5. Adjustment of Structural Parameters

Generally, the performances of PCF voltage sensors can be modified by changing their geometrical parameters. The impacts of the geometrical parameters d_1_ and d_2_ on sensing performance are discussed when the ambient voltages are 465 and 525 V. Moreover, the influences of the spacing of the triangular-lattice distribution Λ on the sensing performances are also discussed for voltages of 465 and 525 V.

### 5.1. Adjustment of Diameters d_1_ and d_2_

**Figure 7:**
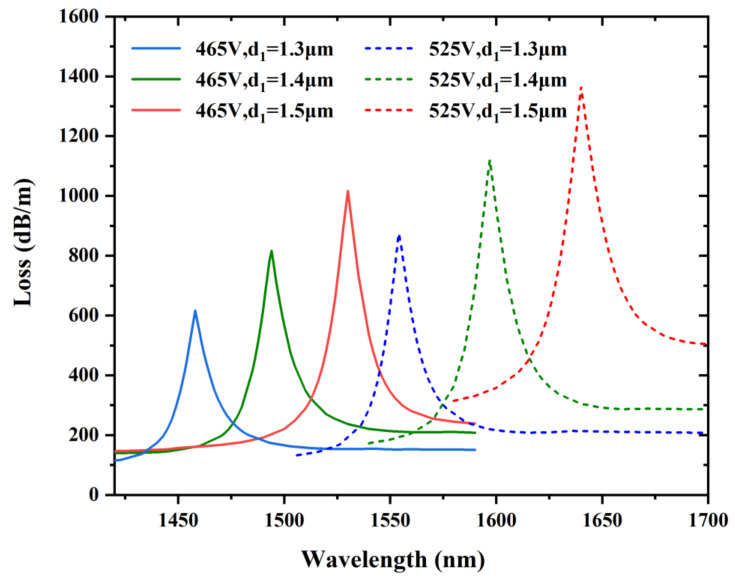
Variation in the confinement loss at the voltages of 465 and 525 V by varying the LC filled hole diameter d_1_ = 1.3, 1.4, and 1.5 μm, while other structural parameters are d = 1.4 μm, d_2_ = 2.2 μm, and Λ = 3 μm.

Figure 7 portrays the variation in the confinement loss with the changes in d_1_ while d = 1.4 μm, d_2_ = 2.2 μm, and Λ = 3 μm which stay unchanged as ambient voltages are 465 and 525 V. It is clearly observed that the resonance wavelength moves to the longer wavelength when the diameter d_1_ is 1.3, 1.4, and 1.5 μm. That is because the RI of the LC defect mode becomes larger while the RI of the fiber mode stays unchanged; thus, achieving a red shift at resonant wavelengths. More energy couples to the LC defect mode for the reason that the LC obtains a greater RI than the background material silica; therefore, confinement losses rise significantly with the increase in the diameter d_1._ In addition, the confinement losses rise on account of the increase in the ambient voltage from 465 to 525 V. At the same time, we can notice that the sensitivity becomes progressively greater with the rising diameter d_1_. We can conclude that the structural parameter d_1_ has a significant influence on the sensitivity of this voltage sensor and we could realize better sensing performance by modifying the diameter d_1_ of the LC filled hole.

**Figure 8:**
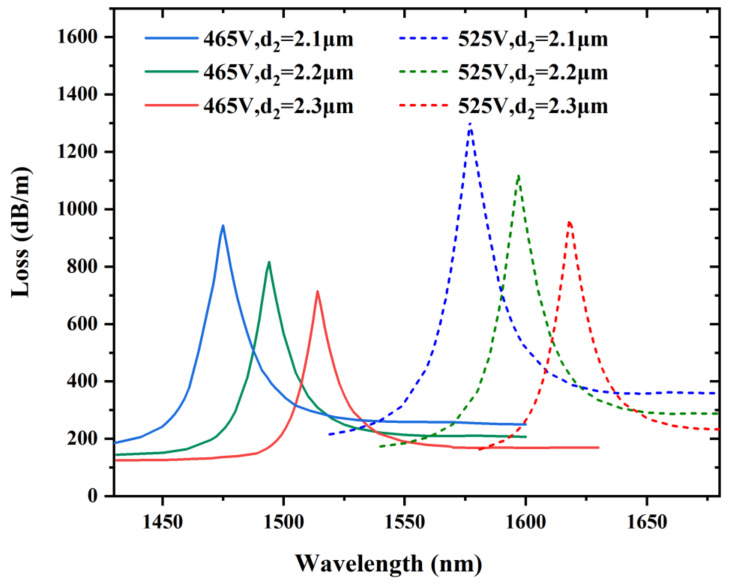
Variation in the confinement loss at the voltages of 465 and 525 V by varying the diameters of the two bigger air holes in the x-direction and d_2_ = 2.1, 2.2, and 2.3 μm, while other structural parameters are d = 1.4 μm, d_1_ = 1.4 μm, and Λ = 3 μm.

The influence of varying the diameter d_2_ of larger air holes in the x-orientation on the resonant wavelength and losses are analyzed. It is obviously illustrated in Figure 8, in which the diameter d_2_ is modulated as 2.1, 2.2, and 2.3 μm. Other structural parameters are represented as d = 1.4 μm, d_1_ = 1.4 μm, and Λ = 3 μm. The resonant wavelength is shifted in the direction of the longer wavelength as the diameter d_2_ expands, but this wavelength shift is not as far as that of modifying the diameter d_1_. This is due to the fact that RI of the fiber mode decreases but that of the LC mode remains unchanged, causing the coupling point to red shift. The confinement losses decrease since less energy is transferred into the microstructure cladding due to the increased diameter d_2_ of the two larger air holes. The sensitivity increases slightly as we increase the diameter d_2_. Hence, we cannot improve the sensing performance significantly by modulating the diameter d_2_.

### 5.2. Adjustment of the Spacing Λ

**Figure 9:**
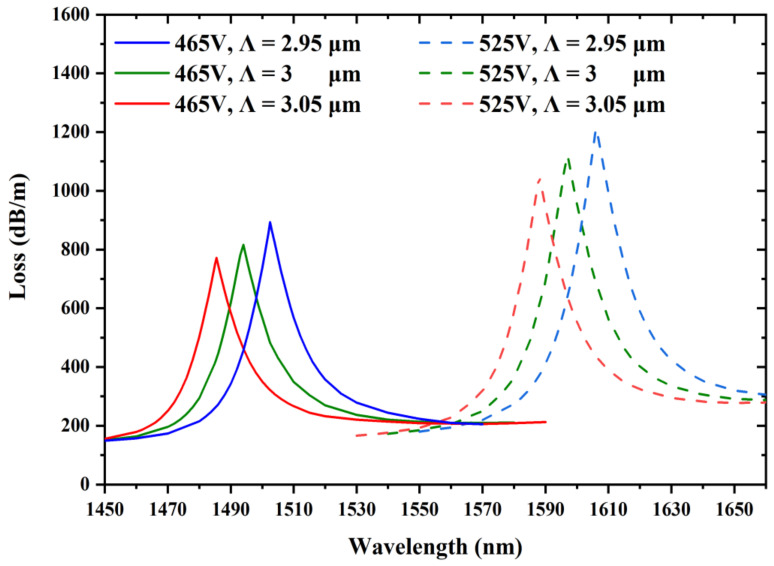
Variation in the confinement loss at the voltages of 465 and 525 V by varying the spacing of the triangular-lattice distribution Λ = 2.95, 3, and 3.05 μm, while other structural parameters are d = 1.4 μm, d_1_ = 1.4 μm, and d_2_ = 2.1 μm.

The influence of changing the spacing of the triangular-lattice distribution Λ on the sensing performance is discussed for the voltages of 465 and 525 V. Other construction parameters are maintained as d = 1.4 μm, d_1_ = 1.4 μm, and d_2_ = 2.1 μm. As shown in Figure 9, it causes a blue shift in the resonant wavelength and confinement loss decreases with the spacing Λ increasing. As a consequence of the index of the fiber mode rising as well as that of the LC defect mode declining, the resonant wavelength shifts in the direction of the shorter wavelength. As spacing Λ becomes larger, less energy couples from the fiber mode to the LC defect mode, resulting in a decrease in losses. It is noticeable that the sensitivity stays essentially unchanged as the spacing Λ changes.

## 6. Conclusions

In this paper, a mode-coupling voltage sensor based on LC-filled photonic crystal fiber was presented and analyzed based on the finite element method. The characteristics of LC vary with different ambient voltages. By calculating and analyzing the confinement loss and the shift of the resonant wavelength as the voltage ranges from 365 to 565 V, the sensor reached a sensitivity of 1.29977 nm/V at the temperature of 25 °C. One hole of the PCF was infiltrated with ethanol which was merely sensitive to temperature to realize the temperature compensation. The standard deviation of the wavelength difference was less than 2 nm within the temperature adjustment from 25 to 50 °C. Moreover, we tried to adjust the relevant structural parameters for better sensing performance. In conclusion, the proposed temperature-compensated voltage sensor is promising in the voltage sensing field and possesses good prospects for applications.

## Figures and Tables

**Table 1:** Parameter values of nematic LC E_7_.

Parameters	Δn (Birefringence)	Δε (Anisotropy)
Values	0.227 (632.8 nm; 20 °C)	12.7 (25 °C; 1 kHz)

## Data Availability

Not applicable.
